# Risk Stratification for the Prediction of Skeletal-Related Events in Patients With Bone Metastases From Non-small Cell Lung Cancer

**DOI:** 10.7759/cureus.95808

**Published:** 2025-10-31

**Authors:** Yoshihiro Sakamoto, Eiji Nakata, Masanori Hamada, Yoshimi Katayama, Shinsuke Sugihara, Toshifumi Ozaki

**Affiliations:** 1 Department of Orthopedic Surgery, Okayama University Graduate School of Medicine, Dentistry and Pharmaceutical Sciences, Okayama, JPN; 2 Department of Orthopedic Surgery, Shikoku Cancer Center, Ehime, JPN

**Keywords:** anaplastic lymphoma kinase, bone metastases, epidermal growth factor receptor-tyrosine kinase, lactate dehydrogenase, non-small cell lung cancer, skeletal related events

## Abstract

Skeletal-related events (SREs) frequently occur in patients with bone metastases from non-small cell lung cancer (NSCLC). This study aimed to identify risk factors for SREs in patients with NSCLC. Based on these factors, we also aimed to stratify patients into subgroups to facilitate the assessment of SRE risk. This retrospective analysis used medical records of 139 patients with NSCLC bone metastases who received treatment at our institution between 2011 and 2014. The incidence of SREs was assessed, and SRE-free survival was analyzed using the Kaplan-Meier method. Clinical information collected at registration was assessed to identify factors associated with the onset of SREs within six months. Univariate analysis was performed using Fisher’s exact test, and multivariate analysis was performed using Cox regression. Of the 139 patients, 36 (26%) developed SREs after registration. The SRE-free survival rates were 80% and 64% at 6 and 12 months, respectively. The univariate and multivariate analyses revealed that the absence of epidermal growth factor receptor (EGFR) mutations or anaplastic lymphoma kinase (ALK) rearrangement (hazard ratio (HR): 4.51, 95% confidence interval (CI): 1.32-15.7, p = 0.017) and a lactate dehydrogenase (LDH) level ≥400 U/L (HR: 8.08, 95% CI: 1.78-36.6, p = 0.0067) were risk factors for SRE presentation within six months. Patients were classified into the following three subgroups: with EGFR mutation or ALK rearrangement and LDH level <400 U/L; without EGFR mutation or ALK rearrangement and LDH level <400 U/L; with/without EGFR mutation or ALK rearrangement and LDH level ≥400 U/L. The corresponding six-month SRE-free survival rates were 92%, 69%, and 34%, respectively, showing significant differences (p < 0.001). Close monitoring is recommended for patients with LDH levels ≥400 U/L in daily clinical practice, particularly with the help of the proficiency of orthopedic and radiological experts, to prevent complications such as pathological fractures and paraplegia.

## Introduction

Bone metastases commonly occur in patients with advanced non-small cell lung cancer (NSCLC) and have been reported in approximately 20-40% of patients with metastatic lung cancer [1-5]. Bone metastases progress gradually and often lead to skeletal-related events (SREs), such as pathological fractures, malignant spinal cord compression (MSCC), the need for bone radiation therapy (RT) or surgery, and malignancy-associated hypercalcemia [1-4].

Despite improved first-line treatments for lung cancer, SREs adversely affect the clinical course in many cases. Approximately 38-65% of all patients with bone metastases from NSCLC experience at least one SRE, whereas 5-31% experience multiple SREs. The incidence rates of radiation, fracture, surgery, MSCC, and hypercalcemia are 30-56%, 3-26%, 1-5%, 3-25%, and 1-3%, respectively [3-8].

Advances in systemic treatment have led to improved survival in patients with advanced NSCLC; however, this has concurrently increased the incidence of bone metastases and extended the timeframe during which patients are susceptible to SRE [1-3]. These complications cause significant skeletal morbidity, reduce quality of life (QOL), and shorten survival durations [9-11]. In particular, patients with MSCC may experience paralysis and QOL deterioration through compromised limb movement, as well as urinary and bowel function [9-11]. Moreover, SREs in patients with NSCLC pose a substantial economic burden. Consequently, early detection and treatment of SREs have become increasingly important in the management of NSCLC.

Evaluation of SRE risk facilitates more accurate monitoring of bone metastases. Patients identified as being at high risk of developing SREs may benefit from timely multidisciplinary interventions, including orthopedic procedures and radiotherapy, initiated early in the decline in activities of daily living (ADL). Such proactive approaches may help prevent or delay fractures and the onset of paraplegia. Therefore, universal and effective biomarkers in NSCLC are critically needed to identify patients at high risk of developing SREs and initiate timely treatment.

Recent investigations have focused on identifying predictors of SREs in patients with NSCLC and bone metastases. The key factors reported include bone metastases at presentation, palliative radiotherapy, previous SREs, male sex, and multiple bone metastases [4,6]. However, these studies included various patient populations, including those without bone metastases and those with known bone metastases, and some had previously experienced an SRE.

Thus far, only two studies have examined patients at the time of diagnosis of bone metastasis [5,7]. Sun et al. reported smoking, non-adenocarcinoma histology, poor performance status (PS) (Eastern Cooperative Oncology Group ≥2), and lack of prior epidermal growth factor receptor-tyrosine kinase inhibitor (EGFR-TKI) therapy as factors associated with SREs. Similarly, da Silva et al. reported associations of SREs with smoking, PS, and multiple bone metastases [7]. However, these studies did not include lactate dehydrogenase (LDH) levels, EGFR mutations, or anaplastic lymphoma kinase (ALK) rearrangements. Based on previous findings indicating that elevated LDH levels and the presence of EGFR mutations are associated with an increased incidence of bone metastases [12,13], we hypothesized that elevated LDH levels, EGFR mutations, or ALK rearrangements may be associated with the development of SREs.

Developing a predictive model for SREs may enable the application of these data to clinical practice. We previously reported that, in patients with bone metastases from breast cancer, a high number of metastatic vertebrae (≥20) and elevated carcinoembryonic antigen (CEA) levels (≥5 ng/mL) were significant risk factors for SREs [14]. Based on the combination of vertebral metastasis count and CEA level, the patients were stratified into four subgroups. Using this risk-stratification model, we identified patients at high risk of SREs who required close monitoring to maintain ADL. However, to our knowledge, no systematic model has been established to predict SRE risk in patients with bone metastases from NSCLC. Therefore, in the present study, we investigated potential risk factors for SREs in this patient population. Furthermore, we developed a predictive model for SREs by combining the identified risk factors and stratifying patients into distinct groups. This risk-stratification model enabled the identification of patients at high risk of SREs who required close monitoring to maintain ADL.

## Materials and methods

Study population

This retrospective study included patients newly diagnosed with NSCLC and radiologically confirmed bone metastases who received treatment at Shikoku Cancer Center, Matsuyama, Japan, between January 2011 and December 2014. Patient records were reviewed retrospectively, and follow-ups were conducted until March 2015. The Human Investigation Committee of the Shikoku Cancer Ethics Committee approved this study (approval number: 2017-26). The requirement for informed consent was waived.

Eligibility criteria

The patients included in this study met the following criteria: histologically or cytologically confirmed diagnosis of NSCLC and stage IV disease with bone metastases or postoperative recurrence. Patients were excluded if they had not undergone a histological or cytological examination, had received treatment for bone metastases prior to enrollment, or had a follow-up period of less than three months. Patients with severe comorbidities that could significantly affect prognosis or interfere with treatment, such as uncontrolled cardiovascular disease, active infections, or severe hepatic/renal dysfunction, were excluded from the study. Patients with stable or manageable comorbidities were included.

Assessment of bone metastases

Diagnostic evaluation comprised computed tomography in 38 cases, 18F-fluoro-2-deoxyglucose positron emission tomography/computed tomography in 104 cases, spinal magnetic resonance imaging in 35 cases, and bone scintigraphy in 56 cases. Bone metastases were assessed by enumerating the distinct osseous sites involved. When several metastatic deposits were present within the same bone, they were collectively considered a single metastatic site.

Assessment of SREs

The incidence of SREs, the first SRE reported post-enrollment, and the risk factors associated with the first SRE observed within six months of enrollment were assessed. SREs referred to the presence of pathological fractures, MSCC, or the initiation of RT or surgical intervention targeting bone metastases. Malignancy-associated hypercalcemia was excluded from this definition because it has no direct effect on ADL. The time to initial SRE was defined as the interval between study enrollment and the first documented SRE during follow-up. Patients who did not experience an SRE during the follow-up period were censored at the time of their last observation. Overall survival (OS) was defined as the duration from enrollment to death from any cause.

Cut‑off value selection of ALP and LDH

The cut-off value for ALP was set at 350 U/L, based on the upper limit of normal (ULN) defined by the JSCC Method, which was used at Shikoku Cancer Center. The cut-off value for LDH was set at 400 U/L based on 2.5 times the ULN. The selection of the LDH cut-off value was informed by prior evidence demonstrating that an increase in LDH levels from 1 to 2.5 times the ULN was associated with poor prognosis in patients treated with chemotherapy or targeted therapies [15].

Statistical analysis

SRE-free survival and OS were evaluated using the Kaplan-Meier method and compared statistically using the log-rank test. To identify risk factors for the first SRE occurrence within six months, the following variables were evaluated at enrollment: age, sex, history of SREs at the time of registration, presence of bone metastases at first presentation, levels of ALP and LDH, number of bone metastases, and presence of EGFR mutations or ALK rearrangement. Univariate analysis was performed using Fisher’s exact test, and multivariate analysis was performed using Cox regression to estimate hazard ratios (HRs) and 95% confidence intervals (CIs). LDH and EGFR mutations, as well as ALK rearrangements, were included in the multivariate analysis. Statistical significance was set at p < 0.05 across all analyses. Outcomes were analyzed using the statistical computing software Bell Curve for Excel version 4.08 (Social Survey Research Information Co., Tokyo, Japan).

## Results

Patient characteristics

In total, 139 patients (69 males and 70 females) were included in this study (Table 1). Of them, 128 had adenocarcinoma, 10 had squamous carcinoma cases, and 1 had large cell carcinoma. The median age was 66 (range: 26-87) years, and the median follow-up duration was 12 (range: 3-50) months. Among patients who succumbed to tumor progression during the observation period (n = 71), the median follow-up duration was 10 months (range: 3-31). Among those who remained alive throughout the follow-up period, the median duration was 13 months (range: 3-50).

**Table 1:** Patient characteristics Continuous variables were reported as median (range), and categorical variables as frequencies (%). EGFR-TKI: epidermal growth factor receptor tyrosine kinase inhibitor, ALK: anaplastic lymphoma kinase

Covariates	Category	Patients, number
Sex	Male	69 (49.6%)
	Female	70 (50.4%)
Age, years	Median (range)	66 (26-87)
Histology	Adenocarcinoma	128 (92.1%)
	Squamous carcinoma	10 (7.2%)
	Large cell carcinoma	1 (0.72%)
Chemotherapy	EGFR-TKI	73 (52.5%)
	ALK inhibitor	6 (4.3%)
	Others	24 (17.3%)

Bone metastases development

Of the total study population, 88 patients (63%) had bone metastases before registration, whereas 51 (37%) did not. Of these 51 patients, 31 were treated for their original lesions before metastases developed (30 underwent resection and one received RT): 18 had synchronous bone metastases and other metastatic lesions, and 13 had metachronous bone metastases that developed during follow-up for other metastatic lesions. The remaining 20 patients presented with stage IV disease without bone metastases, which developed after registration.

Treatments

After registration, 103 of 139 patients received chemotherapy. Among them, 77 had EGFR mutations, of whom 73 received EGFR-TKI therapy. Six patients had ALK rearrangement, and all received ALK inhibitor therapy. The remaining 20 patients predominantly received platinum-based chemotherapy. On the other hand, owing to deterioration in general condition associated with disease progression, the remaining 36 of 139 patients did not receive systemic therapy. Bone-modifying agents (BMAs), including denosumab or zoledronic acid, were administered to 78 patients. In contrast, the remaining 61 patients did not receive BMAs owing to dental issues, renal impairment, or poor general health.

SREs

**Figure 1:**
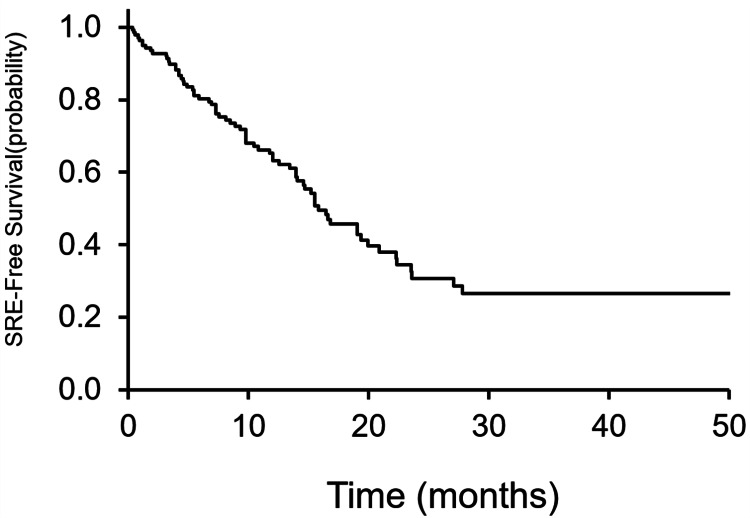
SRE-free survival The SRE-free survival rates at 6, 12, and 24 months were 80%, 64%, and 31%, respectively. SRE: skeletal-related event

SREs were observed in 59 of 139 (42%) and 32 of 139 (23%) patients before recognition of bone metastasis. Among the 88 patients who developed bone metastases before registration, 21 (24%) experienced SREs. Eight of 139 patients (6%) had multiple SREs: five had two SREs, and three had three SREs. The SRE-free survival rates at 6, 12, and 24 months were 80%, 64%, and 31%, respectively (Figure 1).

First SRE after registration

In total, 36 of 139 patients (26%) developed their first SRE after registration. Two of these patients had SREs at multiple locations simultaneously. The most frequently noted location of SREs was the spine (n = 22), followed by the ribs (n = 4), femur (n = 3), humerus (n = 2), scapula (n = 2), clavicle (n = 2), pelvis (n = 2), and skull (n = 1). The SREs were RT (n = 29), MSCC (n = 1), and pathologic fracture (n = 8) (Table 2).

**Table 2:** Location and type pattern of the first SRE SRE: skeletal-related event, RT: radiotherapy, MSCC: malignant spinal cord compression

Location	Count (n)	RT (n)	Pathological fracture (n)	MSCC (n)
Spine	22	19	2	1
Ribs	4	2	2	-
Femur	3	2	1	-
Humerus	2	2	-	-
Clavicle	2	1	1	-
Scapula	2	1	1	-
Pelvis	2	1	1	-
Skull	1	1	-	-

The 22 patients whose first SRE involved the spine comprised two patients with vertebral fracture and one patient with MSCC; RT was required in 19 of these patients. Of the three patients with femoral SREs, one developed a pathological fracture that required operative management, whereas the other two had no fractures and underwent RT. Both patients with SREs involving the humerus without fractures required RT. The median time to first SRE development was 5.7 months (range: 0.3-24). The first SRE occurred within six months of registration in 19 (53%) patients, between 6 and 12 months in seven (19%) patients, and after 12 months in 10 (28%) patients.

Risk factors of first SRE development within six months

Both univariate and multivariate analyses identified the absence of EGFR mutations or ALK rearrangement and LDH level ≥400 U/L as risk factors for first SRE within six months (Table 3): HR 4.51 (95% CI: 1.32-15.7), p = 0.017, and HR 8.08 (95% CI: 1.78-36.6), p = 0.0067, respectively.

**Table 3:** Risk factors for first SRE development within six months Categorical variables were presented as frequencies (%). p-values were derived using Fisher’s exact test. EGFR: epidermal growth factor receptor, ALK: anaplastic lymphoma kinase, ALP: alkaline phosphatase, LDH: lactate dehydrogenase, SREs: skeletal-related events

Covariates	Category	Patients, no.		p-value
		Patients without SREs	Patients with SREs	
Age, years	<65 (n = 65)	56 (86.2%)	9 (13.8%)	1.00
	≥65 (n = 74)	64 (86.5%)	10 (13.5%)	
Sex	Male (n = 69)	59 (85.5%)	10 (14.5%)	0.81
	Female (n = 70)	61 (87.1%)	9 (12.9%)	
EGFR mutation or ALK rearrangement	Yes (n = 84)	77 (91.7%)	7 (8.3%)	0.041
	No (n = 55)	43 (78.2%)	12 (21.8%)	
ALP (U/L)	<350 (n = 92)	82 (89.1%)	10 (10.8%)	0.20
	≥350 (n = 47)	38 (80.9%)	9 (19.1%)	
LDH (U/L)	<400 (n = 129)	115 (89.1%)	14 (10.9%)	0.0046
	≥400 (n = 10)	5 (50.0%)	5 (50.0%)	
Number of bone metastases	1 (n = 64)	58 (90.6%)	6 (9.4%)	0.22
	≥2 (n = 75)	62 (82.7%)	13 (17.3%)	
SREs in bone metastases detection	Yes (n = 32)	26 (81.3%)	6 (18.8%)	0.38
	No (n = 107)	94 (87.9%)	13 (12.1%)	
Bone metastases at presentation	Yes (n = 51)	44 (86.3%)	7 (13.7%)	1.00
	No (n = 88)	76 (86.4%)	12 (13.6%)	

**Figure 2:**
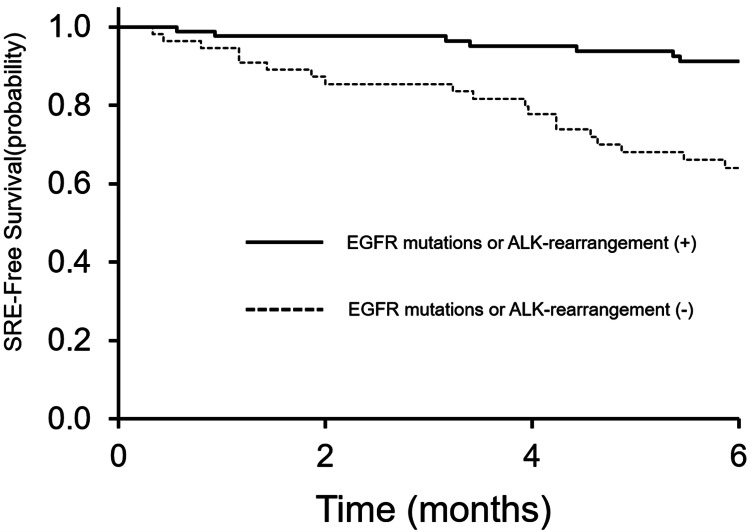
SRE-free survival based on EGFR mutation or ALK rearrangement status The six-month SRE-free survival rates were 91% and 64% in patients with and without EGFR mutations or ALK rearrangements, respectively, with a significant difference (p < 0.001). ALK: anaplastic lymphoma kinase, EGFR: epidermal growth factor receptor, SRE: skeletal-related event

**Figure 3:**
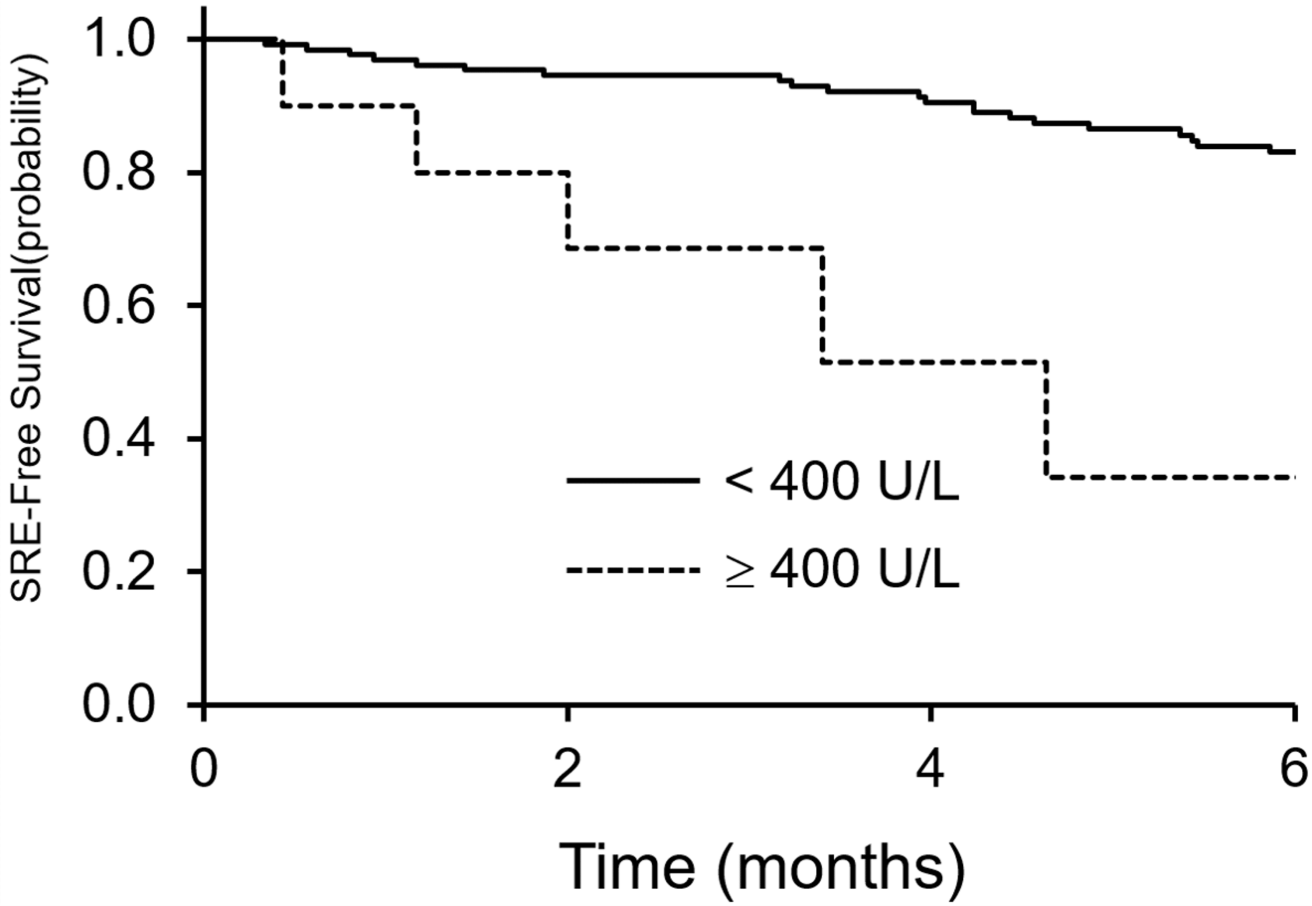
SRE-free survival based on LDH level The six-month SRE-free survival rates were 83% and 34% in patients with LDH levels of <400 U/L and ≥400 U/L, respectively, showing a significant difference (p = 0.021). LDH: lactate dehydrogenase, SRE: skeletal-related event

Only seven of 84 patients (8%) with EGFR mutations or ALK rearrangements developed SREs within six months, compared with 12 of 55 patients (22%) without these changes. Similarly, 14 of 129 patients (11%) with LDH levels <400 U/L developed SREs within six months, compared with 5 of 10 patients (50%) with LDH levels ≥400 U/L. The six-month SRE-free survival rates were significantly different for both factors: 91% and 64% in patients with and without EGFR mutations or ALK rearrangements, respectively (p < 0.001; Figure 2), and 83% and 34% in patients with LDH levels <400 U/L and ≥400 U/L, respectively (p = 0.021; Figure 3).

**Figure 4:**
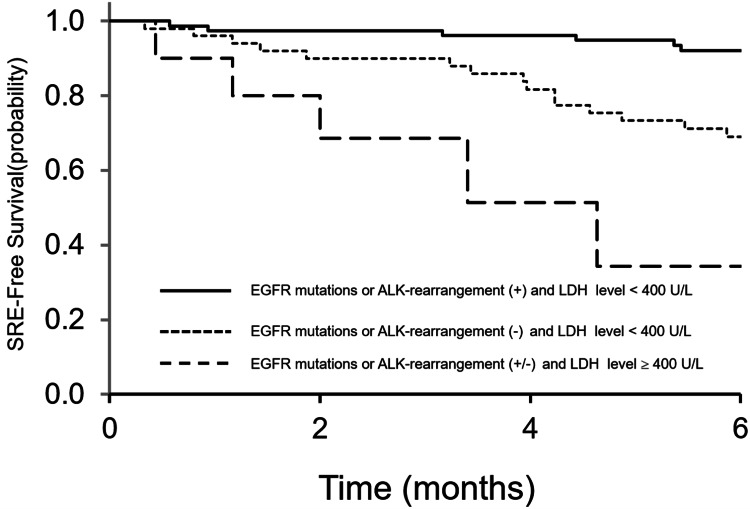
SRE-free survival based on the combination of EGFR mutation or ALK rearrangement status and LDH level Patients were classified into three groups: patients with EGFR mutation or ALK rearrangement and LDH levels <400 U/L; patients without EGFR mutation or ALK rearrangement and LDH levels <400 U/L; and patients with/without EGFR mutation or ALK rearrangement and LDH levels ≥400 U/L. The corresponding six-month SRE-free survival rates were 92%, 69%, and 34%, respectively, showing significant differences (p < 0.001). SRE: skeletal-related event, EGFR: epidermal growth factor receptor, ALK: anaplastic lymphoma kinase, LDH: lactate dehydrogenase

Patients were then classified into three subgroups based on the combination of both factors: with EGFR mutations or ALK rearrangement and LDH levels <400 U/L; without EGFR mutations or ALK rearrangement and LDH levels <400 U/L; and with/without EGFR mutations or ALK rearrangement and LDH levels ≥400 U/L. The corresponding six-month SRE-free survival rates were 92%, 69%, and 34%, respectively, showing significant differences (p < 0.001; Figure 4).

Overall survival

**Figure 5:**
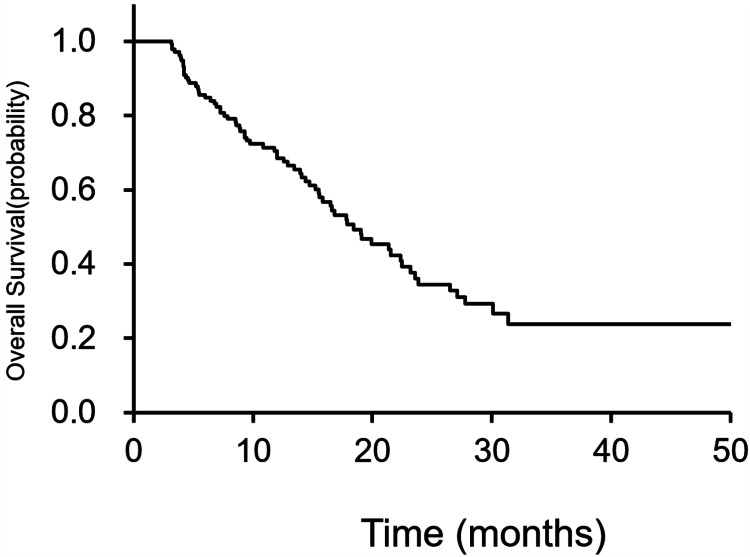
OS OR rates were 85% and 69% at 6 and 12 months, respectively. OS: overall survival

The OS rates at 6 and 12 months were 85% and 69%, respectively (Figure 5).

## Discussion

In this study, SREs were observed in 42% of patients with NSCLC bone metastases. The most frequently observed location was the spine, accounting for 61% (22/36) of patients with the first SRE after registration. Multivariate regression revealed that EGFR mutations or ALK rearrangement and LDH levels ≥400 U/L were risk factors for the development of a first SRE within six months.

EGFR mutations have been reported in approximately 40% of patients with NSCLC in Asian populations [16-18]. For patients with advanced NSCLC harboring EGFR mutations, EGFR-TKIs (afatinib, erlotinib, and gefitinib) are considered the standard first-line treatment [16-18]. Multiple clinical trials have shown that EGFR-TKI therapy yields a higher response rate and improved progression-free survival (PFS) compared with platinum-based doublet chemotherapy in this patient population [16-21]. In phase III clinical trials, EGFR-TKIs showed favorable tolerability and significant clinical efficacy in patients with advanced NSCLC harboring EGFR mutations [19-21]. These studies revealed that EGFR-TKI treatment was associated with an overall response rate (ORR) of 71-83%, a median OS of 21-33 months, and a median PFS of 10-13 months [19-21]. Furthermore, in some studies, gefitinib showed prolonged control of bone metastases [22,23]. Administration of EGFR-TKIs to a patient with NSCLC and mixed bone metastases resulted in a marked reduction in bone pain severity. It was associated with remarkable bone formation in lytic lesions or in patients with negative bone scan findings [22,23].

ALK rearrangement occurs in approximately 3-5% of NSCLC cases [24-26]. ALK inhibitors are the standard first-line treatment, achieving higher response rates and better PFS than platinum-based doublet therapy [24-26]. In phase III studies, ALK inhibitor monotherapy exhibited good tolerance and excellent clinical efficacy, with ORRs of 71-88% and median PFS of 11-35 months [24-26]. In patients with NSCLC and bone metastases, ALK inhibitor use has been associated with significant reductions in bone pain and marked reductions in 99mTc accumulation on bone scintigraphy [27].

In the present study, 95% of patients with EGFR mutations received EGFR-TKI therapy, whereas all patients with ALK rearrangement received ALK inhibitors. These drugs may also be effective against bone metastatic lesions, potentially leading to lower SRE rates in patients with EGFR mutations or ALK rearrangements than in those without.

High LDH levels were also identified as a risk factor for the development of a first SRE within six months and have been consistently linked to poorer outcomes, including shorter PFS and OS, in patients with NSCLC [12,28-30]. A previous meta-analysis suggested that elevated LDH levels were significantly associated with poor PFS and OS in patients with NSCLC treated with EGFR-TKIs [28]. Higher LDH levels indicate disease progression in patients with NSCLC and often indicate tumor progression, particularly in cases of advancing bone metastases and extensive skeletal degradation, which may precipitate SRE onset [12,28-30]. Thus, LDH may serve as a biomarker for predicting SRE onset in NSCLC.

Identifying patients at high risk of developing SREs enables orthopedic surgeons and radiation oncologists to implement appropriate interventions before the onset of pathological fractures or MSCC, thereby helping preserve ADL. Therefore, universal and effective biomarkers are required to identify high-risk patients for early treatment initiation. Various risk factors for SREs have been reported, including bone metastases at initial diagnosis, the number of metastatic bone lesions, baseline serum calcium levels, palliative RT, male sex, and multiple metastatic bone lesions [4-7]. However, in our cohort, the presence of bone metastases at initial diagnosis, their multiplicity, and patient sex did not significantly influence SRE risk. Instead, elevated LDH levels (≥400 U/L) and the presence of an EGFR mutation or ALK rearrangement were associated with SRE occurrence (Table 3).

We compared the incidence both by individual factors and by the combination of LDH ≥400 and EGFR mutations or ALK rearrangement and examined a high-accuracy prediction model. Although the LDH level lacks a unified cutoff value as a biomarker, its inclusion in the predictive model may help with adequate monitoring. Detection of EGFR mutation or ALK rearrangement is used for treatment, leading to earlier diagnosis than when the number of metastases is used. Patients were classified into three groups based on these two factors. The highest survival rate was observed in patients with EGFR mutations or ALK rearrangement and LDH levels <400 U/L (92%), followed by those without EGFR mutations or ALK rearrangement and LDH levels <400 U/L (69%) and those with/without EGFR mutations or ALK rearrangement and LDH levels ≥400 U/L (34%). Our findings suggest that elevated LDH levels (≥400 U/L) at the time of metastatic onset may be associated with a higher risk of bone metastases, warranting vigilant clinical surveillance in such patients. However, the prognostic model developed in this study remains exploratory and requires further validation before it can be applied in routine clinical practice. Therefore, its clinical utility should be interpreted with caution.

BMAs are known to prevent SREs and provide analgesic effects in patients with lung cancer and bone metastases. BMA administration is recommended upon the diagnosis of bone metastasis. In this retrospective study, BMAs were not administered to all patients at the time of bone metastasis diagnosis; in some cases, administration began after the onset of SREs. This suggests that the timing of BMA initiation and potential selection bias may have influenced the results. Therefore, BMA administration was excluded from the final analysis. Further multicenter prospective studies are warranted to clarify the impact of BMA use.

PS is also a clinically important factor. However, in this retrospective study, PS data were incomplete due to missing entries in electronic medical records and variable timing of assessments. Since disease progression and bone metastases can cause pain and affect PS, the lack of consistent, complete data led us to exclude PS from the analysis. Future multicenter prospective studies with standardized PS assessment are needed to address this limitation.

This study has some limitations. First, it was a retrospective, single-center analysis that included a relatively small patient population. The small number of events limits the robustness of the Kaplan-Meier method, the log-rank test, and the Cox regression model, leading to wide CIs and reduced statistical significance. Future prospective investigations with larger sample sizes across multiple institutions are required to validate the findings. Second, many patients succumbed to lung cancer progression before SRE onset and were consequently censored. Although patients followed for less than three months were excluded, selection bias cannot be ruled out. Thus, further research on risk factors for SREs is required, with validation through analyses of independent data sources. Third, the follow-up period for SRE-free survival was relatively short, which may have led to underestimation of late-onset events. A longer follow-up is needed to assess long-term outcomes better. Fourth, the patients were not stratified by PS, and data regarding metastatic sites beyond the bone were omitted, which may have affected the findings. Fifth, not all patients received BMAs, which may have influenced the results. Sixth, although most patients with EGFR mutations or ALK rearrangements received chemotherapy after registration, not all could be treated with it.

## Conclusions

SREs were observed in 42% of patients with NSCLC bone metastases. Those with LDH levels ≥400 U/L may be at a higher risk of developing SREs and should undergo close monitoring of bone metastases to prevent significant morbidity and ensure QOL maintenance. In addition, administration of targeted therapies such as EGFR-TKIs and ALK inhibitors may be protective against bone metastases, potentially decreasing SRE rates in patients with EGFR mutations or ALK rearrangements. Prospective, multicenter studies with larger cohorts are warranted to validate these findings and develop predictive models to inform early interventions for SRE prevention in NSCLC.
